# In Vitro Antiviral and Anti-Inflammatory Activities of *N*-Acetylglucosamine: Development of an Alternative and Safe Approach to Fight Viral Respiratory Infections

**DOI:** 10.3390/ijms24065129

**Published:** 2023-03-07

**Authors:** Magda Marchetti, Barbara De Berardis, Irene Bigioni, Alessia Mariano, Fabiana Superti, Anna Scotto d’Abusco

**Affiliations:** 1 National Centre for Innovative Technologies in Public Health, National Institute of Health, Viale Regina Elena 299, 00161 Rome, Italy; 2 Department of Biochemical Sciences, Sapienza University of Rome, 00185 Rome, Italy

**Keywords:** viral respiratory infections, Influenza A virus, adenovirus, inflammation, *N*-acetylglucosamine, nanotechnology

## Abstract

Viral respiratory tract infections (RTIs) are responsible for significant morbidity and mortality worldwide. A prominent feature of severe respiratory infections, such as severe acute respiratory syndrome coronavirus 2 (SARS-CoV-2) infection, is the cytokine release syndrome. Therefore, there is an urgent need to develop different approaches both against viral replication and against the consequent inflammation. *N*-acetylglucosamine (GlcNAc), a glucosamine (GlcN) derivative, has been developed as an immunomodulatory and anti-inflammatory inexpensive and non-toxic drug for non-communicable disease treatment and/or prevention. Recent studies have suggested that GlcN, due to its anti-inflammatory activity, could be potentially useful for the control of respiratory virus infections. Our present study aimed to evaluate in two different immortalized cell lines whether GlcNAc could inhibit or reduce both viral infectivity and the inflammatory response to viral infection. Two different viruses, frequent cause of upper and lower respiratory tract infections, were used: the H1N1 Influenza A virus (IAV) (as model of enveloped RNA virus) and the Human adenovirus type 2 (Adv) (as model of naked DNA virus). Two forms of GlcNAc have been considered, bulk GlcNAc and GlcNAc in nanoform to overcome the possible pharmacokinetic limitations of GlcNAc. Our study suggests that GlcNAc restricts IAV replication but not Adv infection, whereas nano-GlcNAc inhibits both viruses. Moreover, GlcNAc and mainly its nanoformulation were able to reduce the pro-inflammatory cytokine secretion stimulated by viral infection. The correlation between inflammatory and infection inhibition is discussed.

## 1. Introduction

Respiratory tract infections (RTIs) are very frequent in the population and can cause diseases ranging from the common cold to severe respiratory syndromes. RTIs are a major cause of high morbidity and mortality worldwide, particularly in conditions such as elderly or immunocompromised patients [1,2]. Infections of the upper RT, such as common cold, laryngitis, pharyngitis, otitis and sinusitis, are typically caused by viruses and bacteria [3]. Common cold is a viral disease considered as the most frequent infection in humans, which can be caused mainly by rhinovirus, coronavirus, parainfluenza virus, and adenovirus [4]. Adenovirus (Adv), a major cause of respiratory tract disease in both pediatric and adult patients, generally causes mild infections but severe respiratory manifestations have been linked to pneumonia, bronchitis and chronic obstructive pulmonary disease (COPD) exacerbations [5,6]. Moreover, it has been associated with outbreaks and epidemics in a variety of patient populations, with significant morbidity and mortality [7]. Severe diseases have been observed in association with Adv types 1–7 [8,9]. Cidofovir, the only licensed drug for Adv infections [10], has proven to be ineffective for the treatment of severe adenovirus pneumonia in immunocompetent patients [11] and the high risk of nephrotoxicity limits its use and the duration of therapy in high-risk or immunocompromised patients [12]. Furthermore, no vaccine against Adv has been authorized up to now [10].

Influenza viruses, one of the most common causes of acute respiratory infections, can simultaneously infect both the upper and the lower respiratory tract (RT) [13,14]. Acute viral infections of the lower RT represent a serious health problem for both the pediatric and the adult population and, in industrialized countries, they are the main cause of hospitalization in children under the age of 5 and can even cause death in newborns [15]. The importance of acute respiratory diseases is reflected not only in the morbidity and mortality associated with the acute phase, but also, and above all, in the long-term consequences, especially when lower RTIs are recurrent. Early life factors play a fundamental role in the later onset of chronic respiratory diseases in adulthood [16,17]. Viruses, particularly influenza viruses, are known for their ability to mutate and adapt to new hosts. Clearly the most effective weapon to combat epidemics is a specific vaccine but, as is well known, it takes a long time to develop an effective vaccine towards a new virus and make it available for the entire population. Even with regard to coronavirus disease 2019 (COVID-19), it is still early to know if the vaccines that have been developed so far will be successful in stopping severe acute respiratory syndrome coronavirus 2 (SARS-CoV-2) circulation, as we all hope [18]. Measures of social containment and distancing are certainly useful, even if not always easy to apply, and antiviral drugs associated with symptomatic therapies can contain the spread of the infection and save many human lives in the event of major viral mutations or while waiting for the production of effective vaccines. Therefore, it has clearly emerged that there is a need and urgency to generate new antiviral approaches, which are indispensable in the pre-vaccination period, and can be useful in people who, for various reasons, including the unavailability of vaccines, cannot be vaccinated.

An inflammatory process is present in all RTIs, the intensity of which depends on numerous variables, including the type of pathogen and its virulence, the host’s immunological status, and whether the infection is acute or chronic [19]. It is well known that both COVID-19 and SARS are characterized by an overexuberant inflammatory response and a prominent feature of severe SARS-CoV-2 infection is the cytokine release syndrome [20]. In general, viral infections of the upper RT can be complicated by inflammation of the mucous membrane lining the paranasal sinuses, acute sinusitis, which causes significant physical symptoms and negatively affects the quality of life. Bronchiolitis, the most common lower RTI in children, is characterized by extensive airway inflammation and is mainly caused by respiratory tract epithelial cell infection with respiratory syncytial virus or other viruses including adenoviruses and influenza viruses [21]. Viral infection can also cause pneumonia, an inflammation of the terminal part of the lungs, the pulmonary alveoli, which leads to an alteration of the exchange of oxygen and carbon dioxide and can have serious and life-threatening consequences. The elderly and children represent the age groups most susceptible to contracting pneumonia, of which is one of the most frequent causes of death in the elderly and the leading cause of infant death worldwide [22,23].

The pandemic infection by SARS-CoV-2 has highlighted the importance of using non-steroidal anti-inflammatory drugs to prevent COVID-19 complications [24]. Moreover, the possibility to combine anti-inflammatory drugs with antivirals currently in use to treat COVID-19 patients has also been suggested [25].

Based on these observations, anti-inflammatory therapy could represent a safe and efficient treatment for respiratory virus infections. In this view, greater attention should be paid toward “safe” products that could represent a first approach against inflammation and, perhaps, viral replication. 

There is evidence that some natural products, such as Glucosamine (GlcN) and *N*-acetylglucosamine (GlcNAc), can meet these needs and, therefore, could be used as an “alternative” or “complementary” therapeutic approach to fight respiratory viral infections [26,27,28,29,30].

GlcNAc, an amino sugar, is an essential component of bacterial and fungal cell walls and the animal cell extracellular matrix [31]. It is commonly employed in food supplements to promote and maintain cartilage and bone joint health. In fact, it has been used as a modulator of experimental rheumatoid arthritis (RA) in mouse models [32] and, approved for its lack of adverse effects, is utilized for the treatment of joint illnesses such as osteoarthritis (OA), degenerative joint disease, cartilage loss, and rheumatic diseases in humans [33]. GlcNAc-containing products can be administered parenterally, orally, transmucosally, and topically. According to the outcomes of these administrations, GlcN and its derivatives greatly improve the protection from joint damage [34,35,36,37]. GlcNAc has also been shown to be a promising inexpensive and non-toxic treatment in inflammatory bowel disease such as ulcerative colitis and Crohn’s disease [38]. Other studies have highlighted the role played by this substance against multiple sclerosis and other autoimmune diseases [39,40,41]. To be effective, GlcN has to be administered at a high dosage, usually 1.5 g/die in OA patients [35]. In this way, the amount of GlcN that reaches the joints is 4 μM (716.8 ng/mL) and the concentration in the plasma is 9.92 μM (1777.6 ng/mL), which means that despite the high administered dosage, the bioavailability at the site of interest is very low [42,43].

Additionally, in in vitro models, chondrocytes must be treated with high GlcN concentrations ranging from 10 to 0.5 mM [44,45]. Based on these observations, the search aimed at finding new forms of glucosamine, which allow for it to be administered in low doses, is a very interesting goal.

As GlcNAc is a highly safe compound, it represents a good candidate for a variety of applications, in particular, drug development. Here, we investigated whether GlcNAc is able to reduce the inflammatory response triggered in infected cells and to protect them against viral-infection-mediated injury. In particular, the effect of GlcNAc in both bulk and nanoparticle form was studied to verify if, through the production of nanoparticles, the biological effect of the substance could be optimized, possibly increasing its therapeutic index.

In this research, two different viruses—frequent causes of upper and lower RTIs—the Influenza A/H1N1 virus (as a model of enveloped RNA virus) and the type 2 human adenovirus (as a model of naked DNA virus) have been used. Since infection with both viruses can trigger severe lung inflammation and induce acute lung injury, limiting viral replication and relieving inflammation are two important therapeutic strategies in fighting these infections.

## 2. Results

### 2.1. Characterization of N-Acetylglucosamine

#### 2.1.1. Characterization of GlcNAc in Bulk Form

In the first series of experiments, GlcNAc in bulk form was analyzed. After suspension in H_2_O Milli-Q, GlcNAc joined into several and large agglomerates. DLS analysis showed high instability, hydrodynamic diameter and polydispersity index (PDI) values equal to 2.5 ± 1.4 µm and 1.2 respectively, indicating a high polydispersion of suspension data.

**Figure 1:**
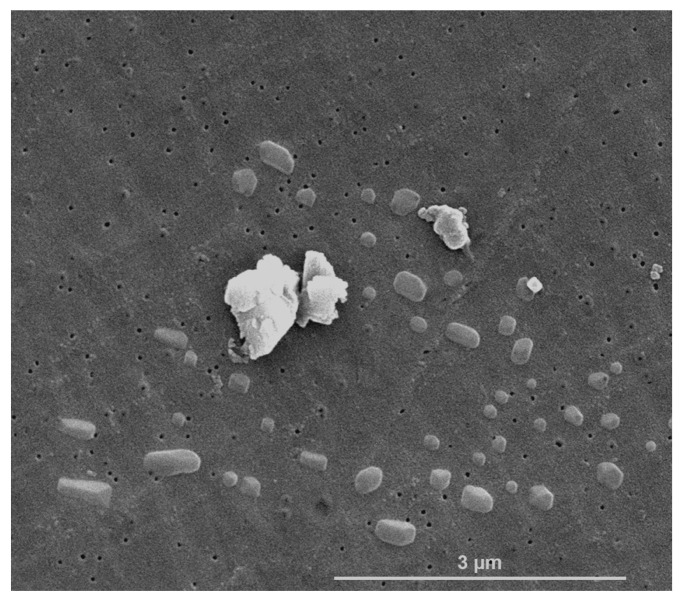
Secondary electron (SE) image obtained by Scanning Electron Microscopy (SEM) analysis showing the principal morphologies of *N*-acetylglucosamine (GlcNAc) particles: irregular shapes, elongated shapes, and agglomerates.

Scanning Electron Microscopy (SEM) analysis of GlcNAc in bulk form allowed for us to detect three principal morphologies: agglomerates with sizes up to 2.5 µm, particles with an irregular shape with an average diameter up to 850 nm, and elongated shape particles with an average diameter up to 300 nm (Figure 1).

**Figure 2:**
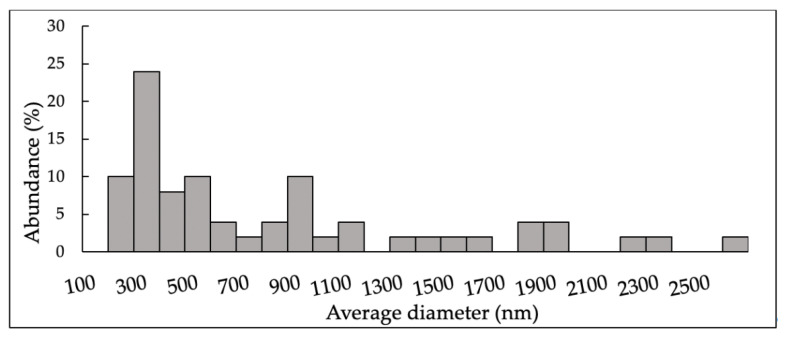
Size distribution of *N*-acetylglucosamine (GlcNAc) particles obtained by Scanning Electron Microscopy (SEM) analysis. The histogram was compiled from a sequence of micrographs.

In Figure 2, the size distribution of GlcNAc particles obtained by SEM analysis is displayed.

A wide range of size distributions (300–2700 nm) was observed, indicating that particles in nanoform were not detectable. 

**Figure 3:**
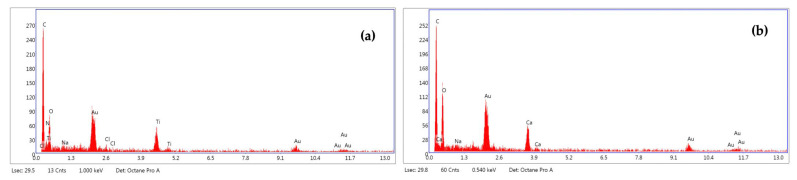
Elemental composition of *N*-acetylglucosamine (GlcNAc) particles rich in: (**a**) C, O, Ti, N, Na, Cl; (**b**) C, O, Ca, Na determined by EDX spectra acquired for each particle analysed by SEM analysis.

Energy Dispersive X-ray (EDX) spectra acquired by GlcNAc particles focused out different impurities in their elemental composition. X-ray microanalysis allowed for us to identify different types of particles: particles rich in C, O with or without Na, Cl traces; particles rich in C, O, Ti with traces of N, Na, and Cl; particles rich in C, O, Ca, Na (Figure 3).

#### 2.1.2. Characterization of *N*-Acetylglucosamine Nanoparticles (GlcNAc-NPs)

**Figure 4:**
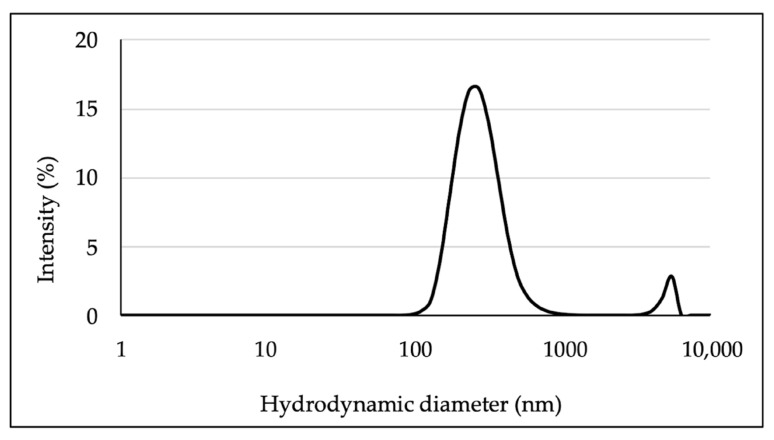
Size distribution by intensity of *N*-acetylglucosamine nanoparticle (GlcNAc-NP) suspensions obtained by DLS analysis. The distribution is the mean of data obtained by three independent experiments.

Suspensions of GlcNAc-NPs in H_2_O Milli-Q at 221 µg/mL concentration were characterised by Dynamic Light Scattering (DLS). Hydrodynamic diameter (Z-Average) and polydispersity index (PDI) values were 312 ± 32 nm and 0.383 ± 0.055, respectively, indicating polydispersion of NP suspension. Size distribution by intensity of GlcNAc-NP suspensions showed one major peak around 267 nm (Figure 4).

Zeta potential of GlcNAc-NPs was −10 ± 3 mV, indicating a negative surface charge of NP and suggesting an instability of suspensions.

**Figure 5:**
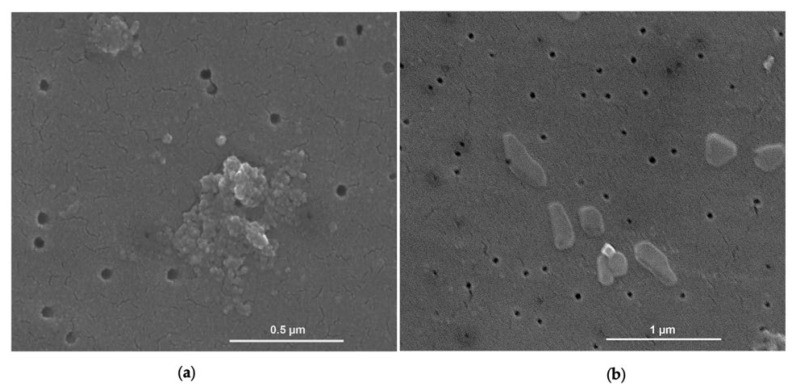
Secondary electron (SE) image obtained by Scanning Electron Microscopy (SEM) analysis showing the principal morphologies of *N*-acetylglucosamine nanoparticles (GlcNAc-NPs): (**a**) single spherical NPs and agglomerates of spherical NPs; (**b**) elongated and irregular NPs.

SEM analysis showed three principal morphologies of GlcNAc-NPs: single spherical particles, agglomerates of spherical particles, and elongated and irregular-shaped particles (Figure 5).

The average diameter of single spherical particles ranged from 21 nm to 90 nm, the agglomerates were in the range of 65–548 nm. The elongated and irregular shape particles had a width ranging from 107 nm to 250 nm and a length from 214 nm to 786 nm. 

**Figure 6:**
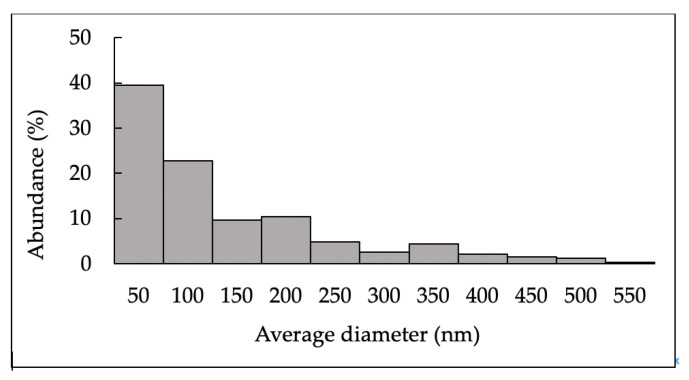
Size distribution of *N*-acetylglucosamine nanoparticles (GlcNAc-NPs) obtained by Scanning Electron Microscopy (SEM) analysis. The histogram was compiled from a sequence of micrographs.

In Figure 6, the size distribution of GlcNAc-NPs obtained by SEM analysis is shown.

The average diameter of the analysed particles was in the range 21–548 nm and 62% of them had an average diameter less than 100 nm.

**Figure 7:**
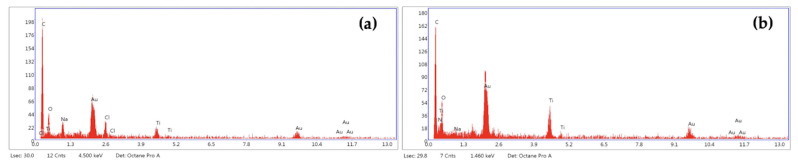

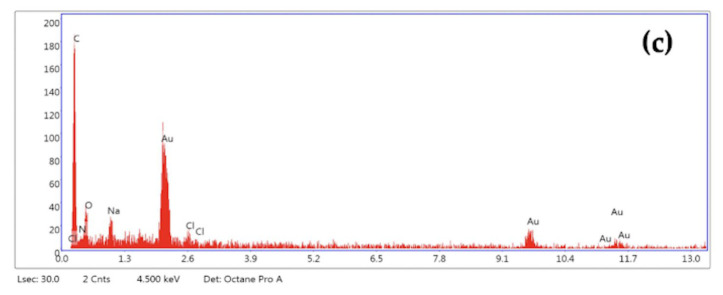
Elemental composition of *N*-acetylglucosamine nanoparticles (GlcNAc-NPs) rich in: (**a**) C, O, Ti, Na, Cl; (**b**) C, O, Ti, N, Na; (**c**) C, O, Na, Cl determined by Energy-dispersive X-ray (EDX) spectra acquired for each particle analysed by Scanning Electron Microscopy (SEM) analysis.

Energy-dispersive X-ray (EDX) spectra acquired by GlcNAc-NPs allowed for us to identify three principal types of particles also detected in GlcNAc bulk form: (1) particles rich in C, O, Ti, Na, Cl; (2) particles rich in C, O, Ti, N; (3) particles rich in C, O, Na, Cl (Figure 7). 

Size distribution obtained by SEM analysis allows for us to consider the sample GlcNAc-NPs a nanomaterial, as indicated by the European Commission definition [46].

### 2.2. Biological Assays

#### 2.2.1. GlcNAc and GlcNAc-NPs Toxicity in Madin-Darby Canine Kidney (MDCK) Cells

**Figure 8:**
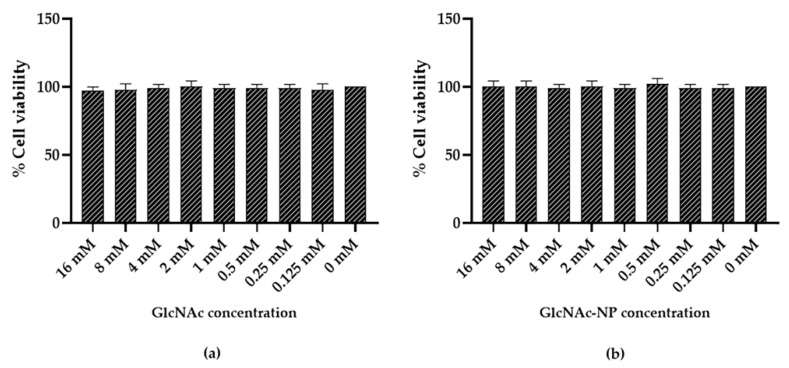
Madin-Darby Canine Kidney (MDCK) cell viability after incubation with *N*-acetylglucosamine (GlcNAc) (**a**) or *N*-acetylglucosamine nanoparticles (GlcNAc-NPs) (**b**). Cell viability was assessed by the 3-(4,5-dimethylthiazol-2-yl)-2,5-diphenyl tetrazolium bromide (MTT) colorimetric method, and MDCK cells were treated with different concentrations of GlcNAc and GlcNAc-NPs (16 mM, 8 mM, 4 mM, 2 mM, 1 mM, 0.5 mM, 0.25 mM, and 0.125 mM) or with culture medium alone (0 mM, cell control) for 72 h. Data represent the means of at least three independent experiments.

To establish the maximal non-cytotoxic dose of GlcNAc and GlcNAc-NPs, two-fold serial dilutions of each substance in serum-free Minimal Essential Medium (MEM) were incubated for 72 h at 37 °C with semi-confluent MDCK cells grown in 96-well tissue culture microplates. Under these conditions, both preparations did not affect cell viability up to the highest dose (Figure 8).

#### 2.2.2. GlcNAc and GlcNAc-NPs Toxicity in A549 Cells 

**Figure 9:**
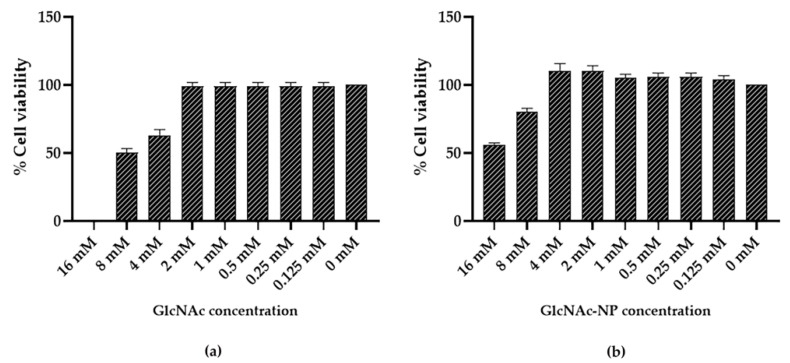
A549 cell viability after incubation with *N*-acetylglucosamine (GlcNAc) (**a**) or *N*-acetylglucosamine nanoparticles (GlcNAc-NPs) (**b**). Cell viability was assessed by the 3-(4,5-dimethylthiazol-2-yl)-2,5-diphenyl tetrazolium bromide (MTT) colorimetric method, and A549 cells were treated with different concentrations of GlcNAc and GlcNAc-NPs (16 mM, 8 mM, 4 mM, 2 mM, 1 mM, 0.5 mM, 0.25 mM, and 0.125 mM), or with culture medium alone (0 mM, cell control) for 72 h. Data represent the means of at least three independent experiments.

To establish the maximal non-cytotoxic dose of GlcNAc and GlcNAc-NPs, two-fold serial dilutions of each substance in serum-free RPMI 1640 medium were incubated for 72 h at 37 °C with semi-confluent A549 cells grown in 96-well tissue culture microplates. As shown in Figure 9, GlcNAc and GlcNAc-NPs were found to be non-toxic in this cell line up to a concentration of 2 mM and 4 mM, respectively.

#### 2.2.3. In Vitro Antiviral Activity of GlcNAc and GlcNAc-NPs towards Influenza A Virus (IAV) Infection

**Figure 10:**
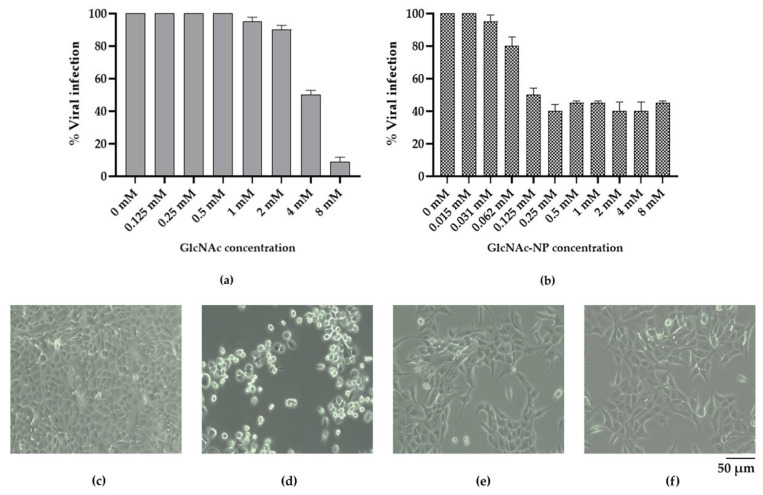
Activity of *N*-acetylglucosamine (GlcNAc) (**a**) or *N*-acetylglucosamine nanoparticles (GlcNAc-NPs) (**b**) against Influenza A virus (IAV) infection. Madin-Darby Canine Kidney (MDCK)-infected cells were treated with different concentrations of GlcNAc (8 mM, 4 mM, 2 mM, 1 mM, 0.5 mM, 0.25 mM, and 0.125 mM) and GlcNAc-NPs (8 mM, 4 mM, 2 mM, 1 mM, 0.5 mM, 0.25 mM, 0.125 mM, 0.062 mM, 0.031 mM, and 0.015 mM) or with culture medium alone (0 mM, virus control) for 72 h. Data represent the means of at least three independent experiments. Panels (**c**–**f**) show light microscopy images of mock-infected MDCK cells (**c**), untreated infected cells (**d**), 4 mM GlcNAc-treated infected cells (**e**), and 0.125 mM GlcNAc-NP-treated infected cells (**f**).

Then, in order to determine the concentration necessary to inhibit viral infection by 50% (effective concentration 50%, EC_50_), the ability of two-fold serial dilutions of GlcNAc and GlcNAc-NPs, starting from 8 mM, to inhibit IAV cytopathic effect in MDCK cells was tested. In these experiments, the two different forms of GlcNAc were present throughout the infection. Under these experimental conditions, GlcNAc showed a dose-dependent inhibitory activity, being able to prevent 50% of IAV cytopathic effect in MDCK cells at 4 mM (Figure 10a), whereas GlcNAc-NPs prevent 50% of infection at 0.125 mM (Figure 10b).

#### 2.2.4. In Vitro Antiviral Activity of GlcNAc and GlcNAc-NPs towards Adenovirus 2 (Adv) Infection

**Figure 11:**
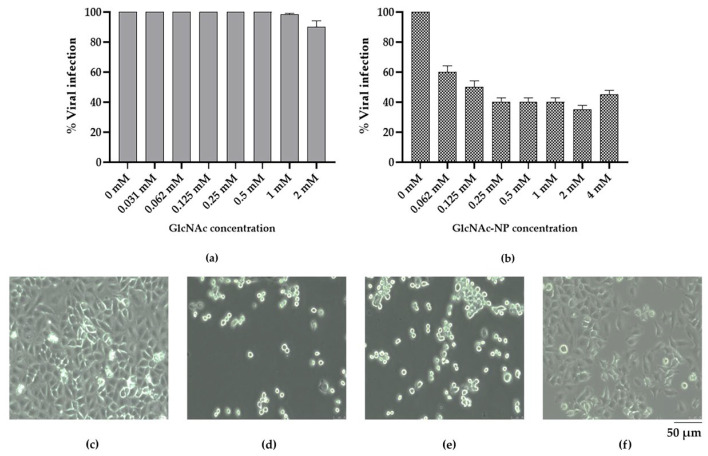
Activity of *N*-acetylglucosamine (GlcNAc) (**a**) or *N*-acetylglucosamine nanoparticles (GlcNAc-NPs) (**b**) against Adv infection. A549-infected cells were treated with different concentrations of GlcNAc (2 mM, 1 mM, 0.5 mM, 0.25 mM, 0.125 mM, 0.062 mM, and 0.031 mM), GlcNAc-NPs (4 mM, 2 mM, 1 mM, 0.5 mM, 0.25 mM, 0.125 mM, 0.062 mM) or with culture medium alone (0 mM, virus control) for 72 h. Data represent the means of at least three independent experiments. Panels (**c**–**f**) show light microscopy images of mock-infected A549 cells (**c**), untreated infected cells (**d**), 2 mM GlcNAc-treated infected cells (**e**), and 0.125 mM GlcNAc-NP-treated infected cells (**f**).

Regarding Adv infection in A549 cells, differently from what was observed for IAV, GlcNAc treatment was unable to prevent viral replication (Figure 11a). Conversely, the concentration of GlcNAc-NPs required to inhibit viral replication by 50% also in this virus-cell system was 0.125 mM (Figure 11b).

#### 2.2.5. Selectivity Index of Different GlcNAc Forms

In order to compare the efficacy of the two different forms of GlcNAc, the selectivity index was determined. Results are reported in Table 1 and Table 2.

As shown in the tables, the selectivity index of GlcNAc in nano form (SI > 128) was higher than that of bulk GlcNAc in both virus-cell systems.

#### 2.2.6. Cytokine Secretion Assay

The effect of the two forms of GlcNAc on the inflammatory process induced by viral infection was investigated. In these studies, the infected cells were treated with the same concentrations of GlcNAc and GlcNAc-NPs (EC_50_) used to determine their SI.

**Figure 12:**
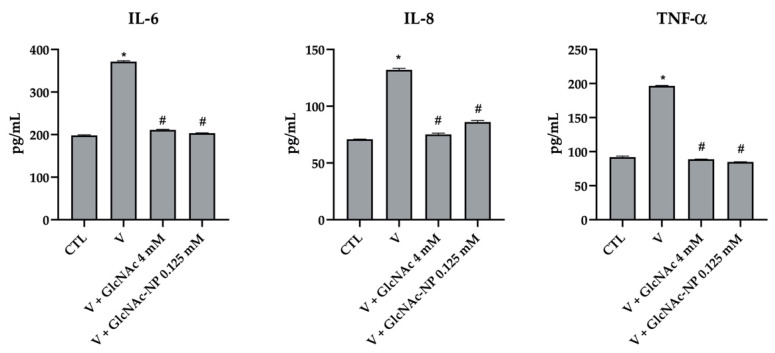
Effects of *N*-acetylglucosamine (GlcNAc) and *N*-acetylglucosamine nanoparticles (GlcNAc-NPs) on IL-6, IL-8 and TNF-α production in Madin-Darby Canine Kidney (MDCK) cells. Cells were exposed to Influenza A virus (IAV) for 72 h or exposed to virus and treated with 4 mM GlcNAc and 0.125 mM GlcNAc-NPs. Then, the amount of cytokine produced was measured in the culture medium of cells and analyzed by ELISA. The results are reported as pg/mL. Results are expressed as mean ± SD of data obtained by three independent experiments. * *p* < 0.05 vs. CTL; # *p* < 0.05 vs. Virus (V).

The production of tumor necrosis factor alpha (TNF-α), Interleukin-6 (IL-6) and Interleukin-8 (IL-8) was determined in the supernatants of MDCK cells 72 h after IAV infection. IAV induced a statistically significant stimulation of all three analyzed cytokines (Figure 12). Interestingly, both 4 mM GlcNAc and 0.125 mM GlcNAc-NPs were able to bring back cytokine secretion to the mock-infected cell level (CTL) (Figure 12). 

**Figure 13:**
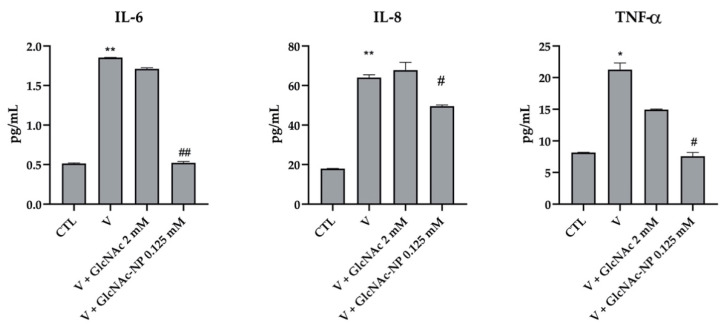
Effects of *N*-acetylglucosamine (GlcNAc) and *N*-acetylglucosamine nanoparticles (GlcNAc-NPs) on IL-6, IL-8 and TNF-α production in A549 cells. Cells were exposed to adenovirus 2 for 72 h or exposed to the virus and treated with 2 mM GlcNAc and 0.125 mM GlcNAc-NPs. Then, the amount of cytokine produced was measured in the culture medium of cells and analyzed by ELISA. The results are reported as pg/mL. Results are expressed as mean ± SD of data obtained by three independent experiments. * *p* < 0.05 vs. CTL; ** *p* < 0.01 vs. CTL; # *p* < 0.05 vs. Virus (V); ## *p* < 0.05 vs. V.

The production of TNF-α, IL-6, and IL-8 was determined also in the supernatants of A549 cell culture at 24, 48, and 72 h after Adv infection. In this experimental system, we additionally found that the virus was able to induce the production of cytokines only at 72 h post-infection. Differently from what observed in IAV-infected MDCK cells, 2 mM GlcNAc was not able to counteract the inflammation stimulated by Adv infection (Figure 13). Conversely, 0.125 mM GlcNAc in nano form was able to bring back IL-6 and TNF-α production to the mock-infected cell level and to partially but significantly reduce IL-8 secretion (Figure 13). As 2 mM GlcNAc was not able to prevent Adv infection (Figure 11), these results suggest a link between a reduction in virus-induced inflammation and an inhibition of viral infection.

## 3. Discussion

Viral respiratory diseases have a high potential for worldwide fast spread. Due to their seasonal circulation, they can cause annual outbreaks of diseases with symptoms ranging from mild to severe and even death. The recent appearance of SARS-CoV-2-related lung infections and the ensuing coronavirus disease 2019 (COVID-19) once again demonstrated the significant risk of contracting viral acute respiratory infections. The main reason for the hospitalization of patients with severe respiratory viral illness is the development of a pulmonary inflammatory disorder, which is connected to lung tissue damage, edema, and the exacerbated inflammatory process [47]. Among the most common respiratory viruses responsible for acute infections, there are Influenza A virus (IAV, an enveloped RNA virus) and adenovirus (Adv, a naked DNA virus). Both these viruses have been linked to pneumonia, causing a significant disease burden in children as well as in adults [37,48,49,50,51]. Moreover, IAV and Adv are also causes of inflammation and it has been observed that the upregulation of inflammatory cytokines during viral infection may promote virus survival and/or the exacerbation of clinical disease [52,53]. It is, therefore, extremely important to target this response in order to reduce host-initiated, self-inflicted damage following infection [54]. In this context, there is growing interest on the development of drugs targeting the immune response to infections.

IAV infection is a major public health threat worldwide, as evidenced by the severe pneumonia caused by this virus each year. Interleukin-6 (IL-6), interleukin-8 (IL-8), together with tumor necrosis factor alpha (TNF-α), are pro-inflammatory mediators that are involved in the onset of inflammation during IAV infection and profoundly contribute to the viral pathogenesis [55,56,57]. In particular, there is a direct relationship between the levels of IL-6 and TNF-α in upper respiratory secretions and the degree of viral replication, fever, and respiratory and systemic symptoms [58]. The production of these two cytokines during IAV infection is associated with symptomatic manifestations [56,59] and it has been observed that the inhibition of IL-6 or TNF-α production protects mice against the severity of IAV infection [60]. 

Adv infection can cause pneumonia and disseminated disease in both immunocompetent and immunocompromised hosts [61]. In particular, Adv is responsible for serious infection in congenitally immunocompromised people, in patients undergoing immunosuppressive treatment for organ and tissue transplants or for cancer, and in human immunodeficiency virus-infected individuals [62]. Adv-induced inflammation is considered to be one of the major causes of severe symptoms [63] and infected alveolar epithelial cells are believed to mediate the inflammatory response to Adv [64]. In particular, some pro-inflammatory mediators, IL-6, IL-8 and TNF-α, have been shown to be elevated upon AdV infection [61,65,66] and both IL-6 and IL-8 have been suggested to contribute to disease severity [63,65]. IL-6 has been demonstrated to play an important role in the acute-phase innate response [67]. Clinical trials using recombinant Adv have also suggested the interconnection between Adv-related cytotoxicity and IL-6 [68,69]. In addition, Qi and coworkers [63] observed that nasal IL-6 levels were higher in inpatients than in outpatients, suggesting a correlation between nasal IL-6 and illness severity. IL-8, an essential mediator of the inflammatory response to different stimuli, including viruses [70], plays a role in the pathophysiology of asthma and obstructive lung disease, as its levels are increased in patients with these chronic inflammatory disorders [71]. In a study carried out on A549 cells infected with Adv type 7 or Adv type 5, it has been observed that Adv type 7 replication was more efficient compared to Adv type 5 replication. Since only Adv type 7 was able to induce IL-8, a direct relationship between specific IL-8 induction and viral replication efficiency has been suggested [72]. 

Consequently, there is growing evidence that pharmacological manipulation of inflammation development may be suitable as a broad new antiviral approach, as the severity of infection seems to depend heavily on these processes (viral replication and pro-inflammatory cytokine induction). Hence, the rebalancing of the inflammatory response to baseline levels represents a potential therapy to restore metabolic homeostasis in the infected host and can possibly mitigate the viral spread and the severity of the infection. 

Based on these observations, anti-inflammatory therapy could represent a safe and efficient treatment for respiratory virus infections. In this view, considerable attention is paid to “safe” products that could represent a first approach against both viral replication and inflammation. There is evidence that some natural products meet these needs, and therefore, could be utilized as “alternative” or “complementary” therapeutic approaches [73].

In this study, we evaluated the effect of *N*-acetylglucosamine (GlcNAc), a naturally occurring substance with anti-inflammatory activity [37], on inflammation and on cytopathic damage induced by viral infection. Indeed, GlcNAc, a pure, safe, and versatile compound, represents a good candidate for a variety of applications, especially drug development. 

Since acute viral infections of the respiratory tract are caused by a variety of viruses very different from each other in terms of structure and replication cycle, in our research, we utilized an enveloped RNA virus (IAV) and a naked DNA virus (Adv) as a model of viral infection. Furthermore, we used a nanotechnological approach to evaluate whether modifying bulk GlcNAc to nano GlcNAc could enhance its activity. We have chosen this approach as it is known that substances in nanoform have different characteristics from the original ones which allow for their successful use in various biomedical fields. Indeed, nanomaterial properties differ significantly from those of the starting materials mainly for increased relative surface area. As a particle’s size decreases, the percentage of surface atoms increases, thus enhancing surface reactivity.

Because catalytic chemical reactions take place on the surface, a given mass of material in nano form will be far more responsive than the same mass of material composed of larger particles. Moreover, having the same size as biological entities, nanoparticles can easily interact with biomolecules on the cell surface and inside the cell.

The results of the viral cytopathic effect reduction test showed that GlcNAc in nanoform had a better IAV inhibitory activity compared to the bulk form. According to the recommendations, the relative safety of both GlcNAc forms has been determined by the selectivity index (SI). In general, a SI of 10 or higher is suggestive of positive antiviral activity, although compounds with a low SI have been also considered as antivirals [74,75,76]. Regarding IAV, the selectivity index of GlcNAc-NPs was found to be higher than that of GlcNAc. Concerning Adv, only GlcNAc-NPs were able to prevent the viral cytopathic effect. The most noteworthy finding was that GlcNAc-NPs have a SI > 128 for both viruses studied. This result confirms that the transformation of a compound from bulk form to nano form results in an improvement of its biological activities. Moreover, as IAV and Adv are unrelated viruses with distinct structures and replication strategies, the observed antiviral activity of GlcNAc-NPs could be attributed to an effect on the cellular machinery involved in the viral replication cycle.

Since GlcNAc is known to have anti-inflammatory activity, we investigated whether it could also indirectly control viral infection outcome by influencing cytokine profiles. Indeed, in addition to the activity against viral infection, both GlcNAc and GlcNAc-NPs also decreased the expression level of IL-6, IL-8 and TNF-α mediators during IAV infection, indicating that it also possesses an anti-inflammatory effect. Interestingly, GlcNAc-NPs were able to reduce virus-induced cytokine secretion at a lower concentration (0.125 mM) compared to GlcNAc (4 mM). Moreover, as expected, only GlcNAc-NPs were able to decrease the expression level of IL-6, IL-8, and TNF-α during Adv infection.

In conclusion, in this research, we demonstrated that GlcNAc and, in particular, GlcNAc-NPs, are safe and effective against IAV. Moreover, GlcNAc in nano form is effective against Adv. Taken together, the findings of our study have provided useful information to counteract IAV and Adv infection and cytopathogenicity through the inhibition of virus-induced inflammation that could favor the development of intervention strategies also through the use of nanotechnological approaches.

To our knowledge, this is the first study showing the potential ability of GlcNAc-NPs to effectively ameliorate the outcome of influenza and adenovirus disease both directly, by inhibition of the viral cytopathic effect, and indirectly, by influencing the inflammatory response. Further evaluation of the GlcNAc-NP mechanism of action would shed light on adequate ways to release the burden caused not only by influenza virus and adenovirus but also by other respiratory viruses that have a similar pathogenesis.

## 4. Materials and Methods

### 4.1. Cells

Madin-Darby canine kidney (MDCK, ATCC, CRL-2936) cells were grown at 37 °C in minimal essential medium (MEM, Invitrogen, Paisley, UK) containing 1.2 g/L NaHCO_3_, and supplemented with 10% inactivated fetal calf serum (FCS, Invitrogen, Paisley, UK), 2 mM glutamine, nonessential amino acids, penicillin (100 IU/mL), and streptomycin (100 μg/mL). 

Hep-2 (human epidermoid carcinoma, larynx, ATCC^®^ CCL-23™) cells were obtained from the American Type Culture Collection (ATCC, Rockville, MA, USA). Cells were grown at 37 °C in a humidified atmosphere with 5% CO_2_ in Minimal Essential Medium (MEM) containing 1.2 g/L NaHCO_3_, and supplemented with 10% inactivated FCS, 2 mM glutamine, nonessential amino acids, penicillin (100 IU/mL), and streptomycin (100 µg/mL) (all from GIBCO, Invitrogen).

A549 (human lung adenocarcinoma cells, ATCC^®^ CCL-185™) cells were obtained from the American Type Culture Collection (ATCC, Rockville, MA) and cultured at 37 °C in a humidified atmosphere with 5% CO_2_ in RPMI 1640 Medium, supplemented with 10% inactivated FCS, 2 mM glutamine, nonessential amino acids, penicillin (100 IU/mL), and streptomycin (100 µg/mL) (all from GIBCO, Invitrogen).

### 4.2. Viruses

The Brisbane-like Influenza A/H1N1 virus strain (IAV) A/RomaISS/02/08 H1N1, kindly provided by Dr. Isabella Donatelli (National Institute of Health, Rome, Italy), was propagated in 80% confluent MDCK cells in serum-free MEM at a multiplicity of infection (m.o.i.) of 1 plaque-forming unit (p.f.u.)/cell. After 60 min of incubation, cells were layered with culture medium supplemented with 0.2% bovine serum albumin fraction V (Sigma-Aldrich, St. Louis, MO, USA) and 1 μg/mL tosyl phenylalanyl chloromethyl ketone (TPCK)-treated trypsin (Sigma-Aldrich, St. Louis, MO, USA). The cells were cultured for 3 to 5 days, cytopathic effect (c.p.e.) was monitored by light microscopy and the virus was harvested when 70% of the cells detached due to c.p.e. Supernatant was withdrawn, centrifuged for 10 min at 1000 rpm, and aliquoted. Virus stocks were stored at −80 °C.

Human adenovirus type 2 (Adv) was grown in HEp-2 cells. The virus was inoculated onto confluent monolayers grown in roller bottles at a multiplicity of infection (m.o.i.) of 1 plaque forming unit (p.f.u.)/cell. After 90 min at 37 °C, the inoculum was removed, and the monolayers were washed once in Phosphate-Buffered Saline (PBS, pH 7.4), incubated at 37 °C in MEM containing 1.2 g/L NaHCO_3_, and supplemented with 2% inactivated FCS, 2 mM glutamine, nonessential amino acids, penicillin (100 IU/mL), and streptomycin (100 µg/mL). When extensive c.p.e. was observed, infected cultures were frozen and thawed three times, centrifuged (3000× *g*; 10 min), and supernatants were stored at −70 °C. This stock virus infectious titer was measured by plaque assay on HEp-2 cells.

### 4.3. Characterization of N-acetylglucosamine (GlcNAc)

*N*-acetylglucosamine (GlcNAc) purchased from Merck (# cat A8625, Merck Life Science, Darmstadt, Germany) and GlcNAc-nanoparticles (GlcNAc-NPs), purchased from Ambiotec di Sergio Ammendola [Via Appia Nord 47, 04012 Cisterna di Latina (LT), Italy], have been characterized. 

#### 4.3.1. Dynamic Light Scattering Characterization

GlcNAc and GlcNAc-NPs, as received by the manufacturer, were suspended in H_2_O and characterized by Zetasizer Ultra instrument (Malvern Instrument, Malvern, UK) in order to determine the hydrodynamic diameter and size distribution. DLS measurements were performed on 1 mL of the suspensions. The equilibration step at 25 °C was set at 2 min. Six determinations were performed on each sample. The instrument software automatically determined the number read and duration of each determination. For the analysis of NP sizes, the data relating to distributions by intensity were examined [77]. For each sample, the mean value of the hydrodynamic diameter (Z-Average) of the NPs and the polydispersity index (PDI) were determined by ZS Xplorer Software 1.2.0.91 (Malvern Instruments, Malvern, UK). 

#### 4.3.2. Zeta Potential

NP sample stability and surface charge were assessed by Zeta potential measurements of GlcNAc-NP suspensions. The measurements were conducted in triplicate on 750 μL of NP suspension using an automatic measurement protocol of Zetasizer Ultra.

#### 4.3.3. Scanning Electron Microscopy/Energy Dispersive X-ray Spectroscopy (SEM/EDX) Characterization

GlcNAc and GlcNAc-NPs were characterized by electron microscopy using scanning electron microscopy (SEM) (FE-SEM Quanta Inspect, FEI Company, Eindhoven, The Netherlands) equipped with a Soft Imaging System to determine the shape, primary size, size distribution, and agglomeration status.

Briefly, 1 mL of GlcNAc or GlcNAc-NP suspensions was transferred on polycarbonate filters with a diameter of 47 mm and a porosity of 50 nm. Portions of filters were mounted on stubs and coated with a gold film by sputtering. Sample analysis was carried out by choosing a beam voltage of 20 KV. For each sample, more than 100 particles were analyzed, and the mean shape and diameter were determined. EDX spectra were also acquired from the analyzed particles in order to determine the elemental composition of both preparations and the presence of any impurities.

### 4.4. Biological Assays

#### 4.4.1. Cytotoxicity Assay

Cytotoxicity assay was performed as reported elsewhere [78]. Briefly, two-fold serial dilutions of each compound in culture medium were incubated at 37 °C with semiconfluent MDCK or A549 cells grown in 96-well tissue culture microplates (Nalge Nunc Europe Ltd., Neerijse, Belgium). After 24, 48, and 72 h, cell morphology was examined by light microscopy then cells were washed in phosphate-buffered saline (PBS, pH 7.4) and 100 μL of MTT solution (0.5 mg/mL in PBS) was added to each well. Following 3 h of incubation at 37 °C, the liquid was carefully withdrawn without touching the sediment or the cells and formazan crystals were dissolved in 100 μL of dimethyl sulfoxide (DMSO). After 15 min incubation at room temperature to ensure that all crystals were dissolved, the plates were read using an ELISA plate reader (PerkinElmer Italia, Monza, Italia) with a 570 nm test wavelength and a 690 nm reference wavelength. Each assay was performed in triplicates and the cytotoxicity was calculated from an average of 3 replicates.

#### 4.4.2. In Vitro Antiviral Activity of GlcNAc and GlcNAc-NPs towards IAV Infection

MDCK cells were seeded in 96-well tissue culture microplates (Nalge Nunc Europe Ltd., Neerijse, Belgium) at the concentration of 20,000 cells/well and allowed to grow for 24 h at 37 °C in 5% CO_2_. After this time, cells were incubated for 1 h at 37 °C with 100 μL/well of virus-GlcNAc or -GlcNAc-NP mixtures or virus alone. Infection was carried out in quadruplicate at a m.o.i. of 0.1. After viral adsorption, cells were rinsed thoroughly and incubated at 37 °C for 72 h in fresh medium in the presence or absence of GlcNAc or GlcNAc-NPs (100 μL/well). The viral cytopathic effect (c.p.e.) was measured by MTT assay and the results were expressed as the percentage of c.p.e. inhibition by comparison with untreated infected control cultures. To calculate the selectivity index and compare the efficacy of the two forms of GlcNAc, the inhibitory titer of GlcNAc or GlcNAc-NPs was reported as EC_50_—the concentration of the substance at which 50% of the MDCK cells were protected from the IAV-induced c.p.e.

#### 4.4.3. In Vitro Antiviral Activity of GlcNAc and GlcNAc-NPs towards Adv Infection

A549 cells were seeded in 96-well tissue culture microplates (Nalge Nunc Europe Ltd., Neerijse, Belgium) at the concentration of 20,000 cells/well and allowed to grow for 24 h at 37 °C in 5% CO_2_. After this time, cells were incubated for 1 h at 37 °C with 100 μL/well of virus-GlcNAc or -GlcNAc-NP mixtures or virus alone. Infection was carried out in quadruplicate at a m.o.i. of 0.1. After viral adsorption, cells were rinsed thoroughly and incubated at 37 °C for 72 h in fresh medium in the presence or absence of GlcNAc or GlcNAc-NPs (100 μL/well). Viral c.p.e. was measured by MTT assay and results were expressed as a percentage of c.p.e. inhibition by comparison with untreated infected control cultures. To calculate the selectivity index and compare the efficacy of the two forms of GlcNAc, the inhibitory titer of GlcNAc or GlcNAc-NPs was reported as EC_50_—the concentration of the substance at which 50% of the MDCK cells were protected from the adenovirus-induced c.p.e.

#### 4.4.4. Cytokine Secretion Assay

Confluent cell monolayers in a 96-well plate grown at 37 °C in 5% CO_2_ were infected with virus-GlcNAc or -GlcNAc-NP mixtures or virus alone, as above described (Section 4.4.2 and Section 4.4.3), for 72 h. Supernatants from mock-infected or virus- infected cells were compared for the expression of IL-6 and TNF-α cytokines and IL-8 chemokine by Enzyme-Linked Immunosorbent assay kits (ELISA Fine Test, Fine Biotech Co., Ltd., Wuhan, China), according to the manufacturer’s instructions. The experiment was repeated twice with similar results in three parallel measurements.

### 4.5. Statistical Analysis

Data from three independent experiments were reported as the mean ± standard deviation (SD). Data were statistically analyzed with two-way repeated-measures analysis of variance (ANOVA) with Bonferroni’s multiple comparison test, using Prism 5.0 software (GraphPad Software, San Diego, CA, USA). *p* value ≤ 0.05 was considered significant.

## 5. Conclusions

In this study, we verified in two in vitro models of viral infection (IAV and Adv) whether a natural anti-inflammatory substance such as GlcNAc could be effective in controlling respiratory viral infections by counteracting both viral replication and the induction of proinflammatory cytokines. GlcNAc was used as bulk form and as NPs, finding that GlcNAc-NPs had better antiviral activity than the bulk form. Furthermore, very interestingly, GlcNAc-NPs resulted in being effective in inhibiting the cytopathic effect of Adv, while bulk GlcNAc resulted in being completely inactive. GlcNAc-NPs also proved to be much more effective in inhibiting the inflammatory activity of both viruses than the bulk form.

In conclusion, GlcNAc-NPs possess anti-IAV and anti-Adv activity and are capable of inhibiting the inflammatory response induced by viral infection. Although we do not yet have in vivo experimental data, the study of the activity of GlcNAc-NPs on IAV and Adv infection and the underlying mechanisms will provide a theoretical and experimental basis to guide the discovery of drugs against these and hopefully other respiratory viruses.

## Figures and Tables

**Table 1:** In vitro anti-Influenza A virus (IAV) efficacy of the two forms of *N*-acetylglucosamine (GlcNAc): comparison of selectivity index values.

GlcNAc Form	CC_50_ (mM)	EC_50_ (mM)	SI
Bulk	>16	4	>4
NPs	>16	0.125	>128

CC_50_: cytotoxic concentration 50%, EC_50_: effective concentration 50%, SI: (selectivity index) = CC_50_/EC_50_.

**Table 2:** In vitro anti-Adv efficacy of the two forms of *N*-acetylglucosamine (GlcNAc).

GlcNAc Form	CC_50_ (mM)	EC_50_ (mM)	SI
Bulk	8	ND	ND
NPs	>16	0.125	>128

CC_50_: cytotoxic concentration 50%, EC_50_: effective concentration 50%, SI: (selectivity index) = CC_50_/EC_50_, ND: not detectable.

## Data Availability

Not applicable.
